# Muro-Neuro-Urodynamics; a Review of the Functional Assessment of Mouse Lower Urinary Tract Function

**DOI:** 10.3389/fphys.2017.00049

**Published:** 2017-02-06

**Authors:** Hiroki Ito, Anthony E. Pickering, Yasuhiko Igawa, Anthony J. Kanai, Christopher H. Fry, Marcus J. Drake

**Affiliations:** ^1^Department of Physiology, Pharmacology and Neuroscience, University of BristolBristol, UK; ^2^Department of Continence Medicine, University of Tokyo Graduate School of MedicineTokyo, Japan; ^3^Department of Pharmacology and Chemical Biology, University of PittsburghPennsylvania, PA, USA

**Keywords:** mouse model, lower urinary tract dysfunction, cystometry, electromyography of the external urethral sphincter, frequency-volume chart

## Abstract

**Background:** Mouse urodynamic tests are fundamental to understanding normal lower urinary tract (LUT) function. These experiments also contribute to our understanding of neurological dysfunction, pathophysiological processes, and potential mechanisms of therapy.

**Objectives:** Systematic assessment of published evidence on urodynamics, advantages and limitations of different urodynamic measurements in mice, and consideration of potential implications for the clinical field.

**Methods:** A search using specific search-terms for urodynamic studies and mice was conducted on PubMed (from inception to 1 July 2016).

**Results:** We identified 55 studies examining or describing mouse neuro-urodynamics. We summarize reported features of mouse urodynamic function deriving from frequency-volume chart (FVC) measurements, voiding spot assays, filling cystometry, and pressure-flow studies. Similarly, an influence of the diurnal cycle on voiding is observed in mice and should be considered when interpreting rodent urodynamic studies, especially FVC measurements and voiding spot assays. Anaesthesia, restraint conditions, or filling rate influence mouse neuro-urodynamics. Mouse cystometric studies have observed intravesical pressure oscillations that accompany urine flow, attributed to high frequency opening and closing of the urethra. This characterization is not seen in other species, except rats. In contrast to human clinical urodynamics, the terminology of these examinations has not been standardized although many rodent urodynamic studies have been described.

**Conclusion:** Mice have many anatomical and physiological similarities to humans and they are generally cost effective, and allow investigation of the effects of aging because of their short lifespan. There are some differences between mouse and human urodynamics. These must be considered when interpreting LUT function in mice, and translational value of murine disease models.

## Background

The neuro-urodynamic control of the lower urinary tract (LUT) requires precise choreography between the central and peripheral nervous systems. These neural mechanisms are complex and impairment are associated with voiding and storage dysfunction; for example, overactive bladder (OAB), urinary incontinence or detrusor underactivity. In clinical practice, human urodynamic studies evaluate the function of the LUT and are useful in the assessment and diagnosis of patients with a broad range of lower urinary tract symptoms (LUTS) (Drake et al., 2016).

A mammalian model is fundamental to understanding LUT function, and these experiments also contribute to our understanding of pathophysiological processes. Rodents have useful anatomical and physiological similarities to humans, and are generally cost effective. Their short lifespan (about 2 years) means it is feasible to evaluate the effects of aging. Rodent urodynamic studies, such as filling cystometry, pressure-flow studies, or neural activity recording, provide crucial information to enable mechanistic insights into the basis of neural control. In the clinical field, the terminology and practice of urodynamic studies have been developed by the International Continence Society (ICS), to standardize such measurements. Given the importance of animal models, a variety of techniques have been adopted to measure pressures in the rodent LUT and proposals made to standardize terminology (Fry et al., 2010; Andersson et al., 2011). Rats are a useful animal model for neuro-urodynamic studies in part because their bladders have been well-characterized by both *in vitro* and *in vivo* experiments.

On the other hand, the urodynamic properties of the mouse LUT have not been characterized as well as those of other rodents. This is possibly because mice are more difficult to handle and the *in vitro/in vivo* properties are more poorly understood (Uvin et al., 2012). However, the ability to generate genetically-modified mice can provide valuable mechanistic insights and therefore it is appropriate to consider mouse urodynamics in the context of current developments. The present review covers recent knowledge of mouse urodynamics and considers potential implications in the clinical field. In addition the limitations of urodynamic measurements in mice are discussed.

## Normal micturition cycle in mice

In neonatal mice, maternal parenting exploits a perineal-to-bladder reflex to trigger voiding. The adult form of voiding triggered by bladder distension does not become functional until several weeks after birth. As the adult reflex appears, the neonatal perineal-to-bladder reflex becomes weaker and eventually disappears (de Groat et al., 1998).

**Figure 1:**
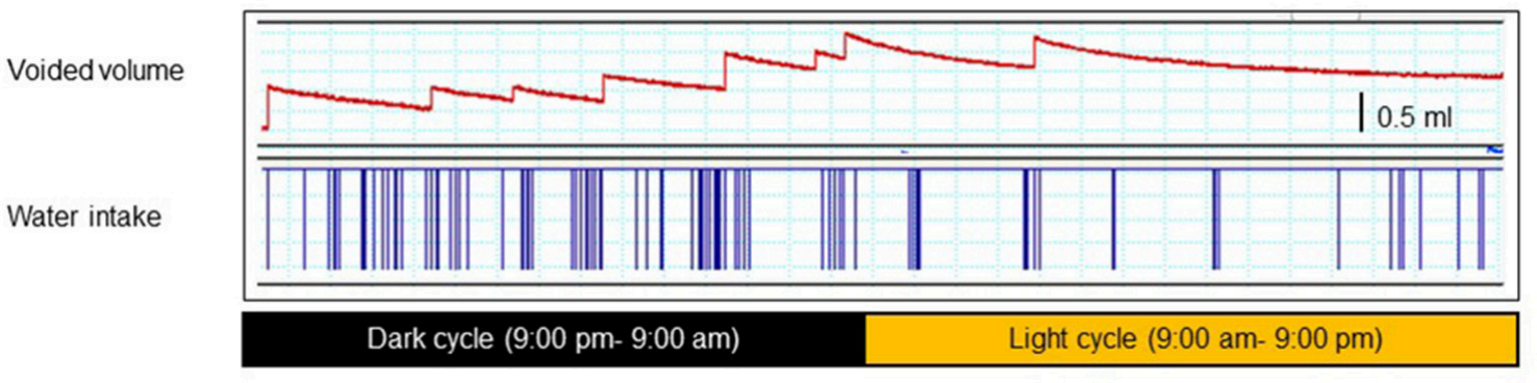
**A 24-h trace of a frequency-volume charts (FVC) (upper) and water intake (lower) of a male mouse at the age of 10 weeks**. Each animal was separately placed in a conscious condition, without any restraint, in a metabolic cage that allows precise measurement of voiding episodes, voided volume, drinking episodes, and amounts (001–006 metMCM/TOA-UFabolic cage, Mitsubishi Chemical Medience, Tokyo, Japan). After 24-h adaptation, voided volume, voiding frequency, and water intake volume were recorded using a PowerLab® data acquisition system continuously for 24 h starting at 9:00 pm. The mice had free access to water and food during recording.

Rodents pass urine with a nocturnal predominance in voiding frequency (Ito et al., 2015; Yoshiyama et al., 2015). Figure 1 is a 24-h trace of frequency-volume chart (FVC) measurements of male mice at the age of 10 weeks and clearly demonstrates that there are notable differences in the voiding behavior and water intake between daytime and night-time. These FVC measurements indicate that young mice (10-weeks old) of both genders usually urinate about 10 times per day, and urination occurs mainly in the dark cycle due, for this nocturnal animal (Aizawa et al., 2013).

Mice are highly social animals and adapt to their surroundings. It is important for precise assessment of mouse voiding behavior to allow for adequate adaptation periods before experiments once animals are moved into a new environment to minimize stress and anxiety. In addition, mice of both sexes use urinary scent marks for territorial communication. Urinary scent incorporates information about individuals (such as species, sex, and individual identity) as well as metabolic information (such as social dominance, and reproductive and health status) (Arakawa et al., 2008; Hou et al., 2016). Voids used for territorial marking seem to be similar to non-territorial voids of mice, and there are no clear criteria to distinguish them *in vivo*.

## Urodynamic techniques available for mice

Given the potential translational relevance of mouse urodynamics, we describe the published evidence in sequence akin to the progressive approach taken in clinical urodynamics (Table 1). Mouse urodynamic function is mainly determined by FVC measurements, voiding spot assays, filling cystometry, and pressure-flow studies. In this section, we summarize the literature for mouse urodynamics, and highlight uncertainties when interpreting their results.

**Table 1:** **Summary of clinical neuro-urodynamic tests for human and relevant mouse neuro-urodynamic tests**.

**Tests**		**Neuro urodynamics**	
		**Human clinical technique**	**Mouse technique**
Non-invasive	Symptom score	Various questionnaires	Not applicable
		such as	
		International Prostate Symptom Score (IPSS)	
		QOL question	
	Bladder diary	Micturition chart	Metabolic cage
		Frequency volume chart (FVC)	Frequency volume chart (FVC)
		Bladder diary	Voiding Spot Assay (VSA)
	Urodynamic tests	Urinary flow rate	Not applicable
		Post-void urine (PVR) analysis	
	Other tests	Near intra-red spectroscopy (NIRS)	Phenotyping (Distended bladder, Perineal for staining)
Invasive	Urodynamic tests	Filling cystometry (storage phase)	Filling cystometry (storage phase)
		Pressure-flow study (voiding phase)	Pressure-flow study (voiding phase)
			Electrophysiology
Future	Special tests	Functional magnetic resonance imaging (fMRI)	Functional magnetic resonance imaging (fMRI)
		Positron emission tomography (PET)	Positron emission tomography (PET)
		Electrophysiology	

### Non-invasive tests (Table 2)

Non-invasive tests may evoke less physical and psycological stress than invasive testing, and allow additional experimental observations to be made. Consequently, it is feasible to undertake longitudinal monitoring of processes like aging or disease progression.

**Table 2:** **Summary of non-invasive tests for mouse urodynamic studies**.

	**Species**	**Sex**	**Weeks**	**Body weight (g)**	**Voided volume per micturition (μl)**	**Day vs. night**	**Reference**
Metabolic cage	C57BL/6J	m	5	16.5 ± 0.3	104.3 ± 7.6	24 h	Aizawa et al., 2013
			9	22.0 ± 0.3	137.1 ± 5.4		
			13	24.3 ± 0.3	177.8 ± 8.6		
			17	25.6 ± 0.2	251.8 ± 15.4		
			21	26.9 ± 0.3	267.4 ± 22.5		
	WT (C57BL/6Cr)	m	8–12	22.1 ± 0.3	about 380	24 h	Yoshiyama et al., 2015
	WT (129Sv and C57BL/6)	f	NA	15–23	490 ± 130	14 h (overnight)	Sutherland et al., 1997
	WT (129Sv and C57BL/6J)	f	12	24.3 ± 0.9	160 ± 30	48 h	Chen et al., 2005
Voiding spot assay	129S1/SvImJ	m	10–12	27.1 ± 0.8	40.1	4 h (9 a.m.–2 p.m.)	Bjorling et al., 2015
		f	10–12	21.8 ± 0.8	103.7		
	C57BL/6J	m	10–12	27.6 ± 0.5	86.7		
		f	10–12	19.2 ± 0.2	68.8		
	NOD/ShiLtJ	m	10–12	29.1 ± 1.3	40		
		f	10–12	24.5 ± 0.4	51.9		
	CAST/EiJ	m	10–12	16.6 ± 0.3	3.8		
		f	10–12	13.4 ± 0.6	15.9		
	WT (C57BL6)	m	9–17	25.7 ± 1.1	NA	1 h	Birder et al., 2002
	C57BL/6	m	NA	20–30	NA	1 h (8–11 a.m.)	Cornelissen et al., 2008
		f	NA	20–30	NA		
	Balb/CAN	m	NA	20–30	NA		
		f	NA	20–30	NA		
	A/J	m	NA	20–30	NA		
		f	NA	20–30	NA		
	B6:129	m	NA	20–30	NA		
		f	NA	20–30	NA		
	129	m	NA	20–30	NA		
		f	NA	20–30	NA		
	WT (C57BL/6 and DBA/2)	f	7–8	27.6 ± 2.25	430 ± 80	overnight 8 h	Jusuf et al., 2001
	C57BL/6	m	10–12	unknown	108.3 ± 14.1	3 h (unknown)	Boudes et al., 2011

*f, female; m, male; NA, not assessed; WT, wild type*.

#### Metabolic cage–frequency-volume chart measurements

Housing mice in metabolic cages enables the measurement of voiding behavior by quantifying the volume of urine drops of freely moving mice (Sutherland et al., 1997; Chen et al., 2005; Aizawa et al., 2013; Yoshiyama et al., 2015). Such FVC measurements using metabolic cages generally allow calculation of voided volume and voiding time, and previous studies have recorded voiding behavior for 14 h (overnight) (Sutherland et al., 1997), 24 h (Aizawa et al., 2013; Yoshiyama et al., 2015), and 48 h (Chen et al., 2005).

Voided volume and bladder capacity are much smaller in mice than in other rodents, so metabolic cages for FVC recording might be less accurate to measure the precise voided volume, because there is a potential problem with evaporation from small urine deposits.

In the FVC measurements, it is important for precise recording of voided volume to remove feces prior to entry into the measuring system. A novel metabolic cage, with a specially-designed net to separate urine from feces, has been recently described (Aizawa et al., 2013; Yoshiyama et al., 2015). This enables recording of precise voided volume, average flow rate, voiding frequency, and water intake of conscious mice continuously for more than 24 h.

As described above, an influence of the diurnal cycle on voiding is observed in mice and should be considered when interpreting rodent urodynamic studies, especially FVC measurements and voiding spot assays (VSA, below), and was achieved in the above papers. To measure precise mouse urinary storage function, mice need a prior adaptation period of more than 24 h.

#### Voiding spot assay (VSA)

VSA is a widely used method to measure the FVC for free moving and awake mice (Birder et al., 2002; Cornelissen et al., 2008; Sugino et al., 2008; Boudes et al., 2011; Yu et al., 2014; Bjorling et al., 2015) and precise measurement of the voiding behavior of mice using a metabolic cage has space and cost implications. The VSA does not allow calculation of urine flow rate, but is useful to measure the pattern and volume of voiding. Voiding behavior is analyzed by placing the animal above or on a piece of filter paper (Jusuf et al., 2001). However, some have indicated that it is difficult to draw inferences about urodynamic parameters from voiding spots, at least in young healthy mice (Bjorling et al., 2015). In fact, some previous VSA studies in mice did not attempt to calculate the voided volume, but only the number of individual spots (Birder et al., 2002; Cornelissen et al., 2008).

The test period and timing in the light-dark cycle are crucial factors in voiding spot assays and FVC measurements, and most papers provide information about when the tests were done (Jusuf et al., 2001; Cornelissen et al., 2008; Yu et al., 2014; Bjorling et al., 2015). Thus, VSAs have been performed in the light phase of the cycle [for 1 h (Cornelissen et al., 2008) and 4 h (Bjorling et al., 2015)], in the overnight period [for 8 h (Jusuf et al., 2001)], but the diurnal phase was not stated in some [for 1 h (Birder et al., 2002) and 3 h (Boudes et al., 2011)].

A modified VSA system, called the automated voided stain on paper (aVSOP) method, has been developed to allow more detailed assessment over an extended time-frame (Negoro et al., 2012). This system has a motor to roll paper under the cage and collect urine stains. This can record accurately the number of voids, voiding frequency, and voided volume for several days.

### Invasive tests

Urodynamic studies in mice include filling cystometry and pressure-flow studies for the investigation of storage and voiding phases, respectively. Although many rodent urodynamic studies have been described, the terminology of these examinations has not been standardized. An absence of reliable terminology risks misinterpretation of the findings and also impedes effective cross referencing between studies and establishment of reference ranges for normality. Therefore, it is important to establish the terminology of rodent urodynamic studies. Only one paper provides a proposed standardization of terminology (Andersson et al., 2011), and this is a suitable reference point for research at the current time.

#### Testing method

Experimental techniques are well-developed and described in several papers (Fry et al., 2010; Andersson et al., 2011). After induction of anesthesia, a catheter is implanted in the bladder dome and tunneled subcutaneously to the abdominal skin incision or head position. The bladder is filled at a controlled rate, and cystometric parameters can be measured via the implanted catheter under non-restrained, restrained, or anesthetized conditions (Andersson et al., 2011; Uvin et al., 2012). In contrast to human clinical urodynamics, where the detrusor pressure is calculated as intravesical pressure minus abdominal pressure, measurement of abdominal pressure is seldom undertaken in mouse urodynamics. One report (Smith et al., 2012) did measure abdominal pressure to calculate detrusor pressure, but in others intravesical pressure is recorded alone (Sutherland et al., 1997; Lemack et al., 2000; Pandita et al., 2000; Jusuf et al., 2001; Birder et al., 2002; Schröder et al., 2003, 2004; Igawa et al., 2004; Chen et al., 2005; Cornelissen et al., 2008; Beamon et al., 2009; Smith and Kuchel, 2010; Soler et al., 2010; Aizawa et al., 2013; Bjorling et al., 2015; Yoshiyama et al., 2015; Franken et al., 2016).

Conscious mouse urodynamic studies have been undertaken under ambulatory or static conditions. Although ambulatory methods may be more physiological catheter problems are common, such as twisting and/or dislodging when using non-restrained mice. However, there are some technical tips to prevent catheter problems, such as securing the catheter to the skin at the point of its external exit, or between the skin and the bladder. Swiveling supports may help to minimize twisting of the tubing. On the other hand, the free-moving status often generates noise and artifacts in the cystometric tracing which can affect accurate analysis. Indeed a previous report indicated that it is difficult to obtain precise voiding volume reliably from awake mice cystometry (Smith and Kuchel, 2010).

Catheters implanted into the bladder in mouse urodynamics generally employ one of two diameters, PE 10 (internal/outer diameter is 0.28/0.61 mm) or PE 50 (internal/outer diameter is 0.58/0.97 mm) tubing. PE 50 is most commonly used for bladder cannulation in mouse urodynamics (Birder et al., 2002; Igawa et al., 2004; Cornelissen et al., 2008; Beamon et al., 2009; Smith and Kuchel, 2010; Boudes et al., 2011; Comiter and Phull, 2012; Smith et al., 2012; Aizawa et al., 2013; Bjorling et al., 2015; Yoshiyama et al., 2015; Franken et al., 2016) and some studies used PE10 for the same purpose (Sutherland et al., 1997; Pandita et al., 2000; Schröder et al., 2003, 2004; Soler et al., 2010; Mingin et al., 2015). PE 10 is thinner and softer than PE 50, which might decrease the physical stress and inflammation caused by catheter implantation in the bladder. However, the small diameter may increase the risk of artifacts during measurements, and potentially failure to measure pressure reliably (Smith and Kuchel, 2010). Although there is a need to compare the merits and disadvantages of the catheter options, the authors were unable to identify any published comparison.

The implantation of a catheter into the bladder dome may cause infection, inflammation or edema in the mucosa and submucosa area, which might influence the cystometric results. In mouse urodynamic studies, catheterization into the bladder is generally done shortly before cystometry. Previous reports, however, show variation in the time interval between catheter implantation and cystometry; 0 day (Sutherland et al., 1997; Lemack et al., 2000; Jusuf et al., 2001; Streng et al., 2002a,b; Boudes et al., 2011; Comiter and Phull, 2012; Smith et al., 2012; Yu et al., 2014; Bjorling et al., 2015; Yoshiyama et al., 2015; Franken et al., 2016), 2 days (Schröder et al., 2003, 2004; Chen et al., 2005; Smith and Kuchel, 2010), 3 days (Pandita et al., 2000; Igawa et al., 2004; Soler et al., 2010; Mingin et al., 2015), 4 days (Aizawa et al., 2013), and 6–8 days (Cornelissen et al., 2008). A longer period after catheter implantation might decrease the post-surgical changes of the bladder; on the other hand, it might increase problems from mucosal regeneration, giving rise to obstruction of the catheter.

Conscious but restrained conditions allow accurate measurements without movement artifacts. In contrast to those in rats, few studies are described in mice and these have been mainly in spinal cord injury models (DePaul et al., 2015; Kadekawa et al., 2016). However, restraint appears to reduce the voided volumes to less than 50 ul (DePaul et al., 2015; Kadekawa et al., 2016). Thus, like anesthesia, restrained conditions might influence urodynamic function (see below).

**Figure 2:**
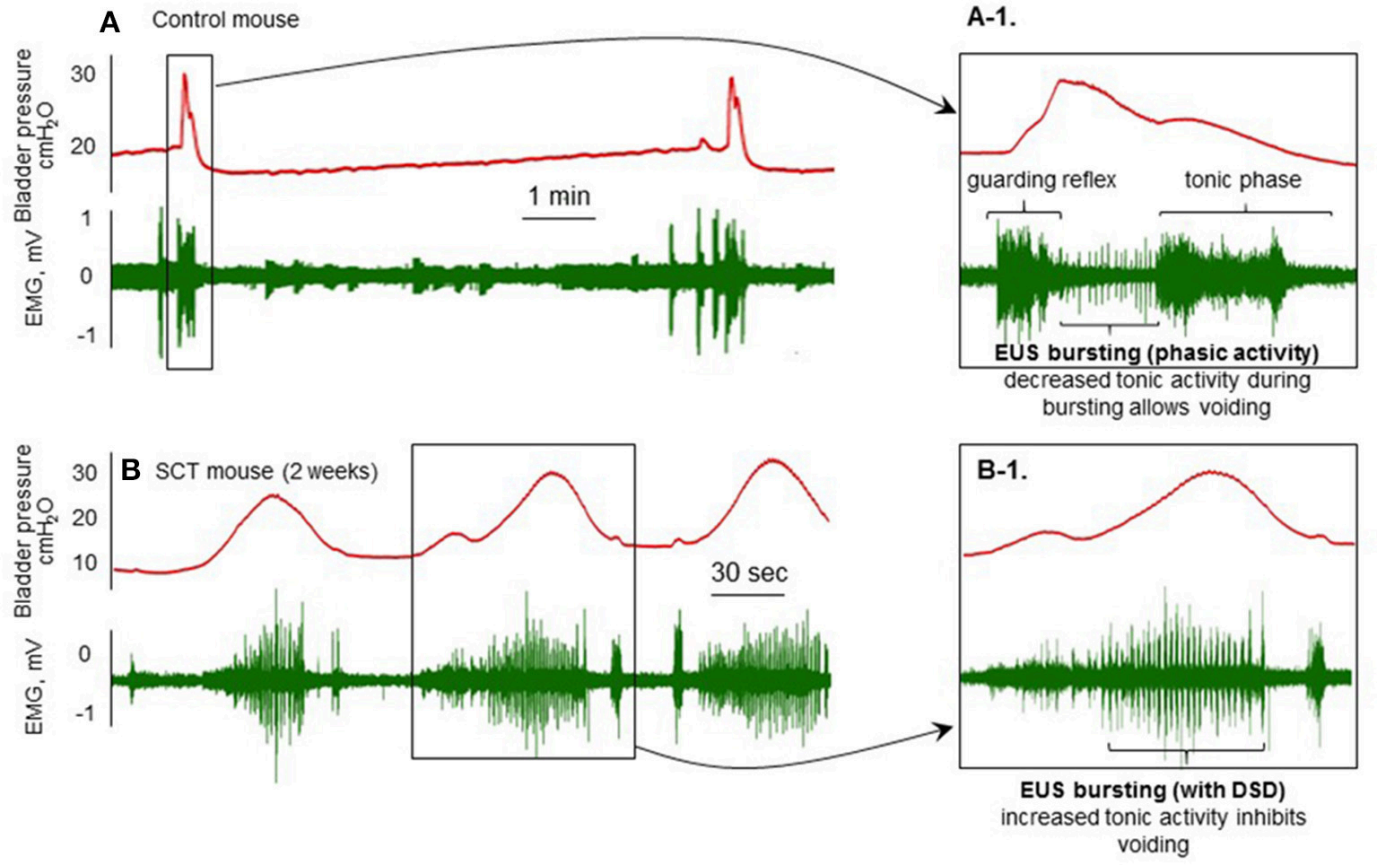
**Cystometry and EMG recordings from spinal cord intact (A)** and T8–T9 spinal cord injury (SCI; **B**) mice 2 weeks post-injury with the brain rostral to the supracollicular level sectioned from the brainstem and removed. Transperineal recording of the external urethral sphincter is combined with conventional filling cystometry. **(A-1)**: This shows the guarding reflex that prevents leaking as bladder pressure approaches threshold. This is followed by increased phasic (bursting) and decreased tonic activity during which voiding occurs and pressure returns to baseline. **(B)**: Following transection and the development of detrusor-sphincter-dyssynergia (DSD; **B-1**), when the bladder contracts, tonic sphincter activity increases resulting in non-voiding contractions and eventually overflow incontinence.

Electromyography of the external urethral sphincter (Figure 2) can be combined with various techniques can be used for conscious restraint (Kadekawa et al., 2016) and decerebration (Sadananda et al., 2011, 2013).

#### Filling cystometry (storage phase)

##### Bladder

Parameters assessed during filling cystometry include bladder capacity, bladder compliance, the number of non-voiding contractions (NVCs), basal pressure (BP) at the initiation of filling and threshold pressure (TP) at onset of micturition. With mouse filling cystometry, the BP and TP are usually around 5—10 cmH_2_O (Pandita et al., 2000; Schröder et al., 2003, 2004; Igawa et al., 2004; Boudes et al., 2011; Aizawa et al., 2013) and 10–40 cmH_2_O (Pandita et al., 2000; Igawa et al., 2004; Schröder et al., 2004; Smith and Kuchel, 2010; Soler et al., 2010; Aizawa et al., 2013), respectively.

Filling rates used vary widely, from 10 (Cornelissen et al., 2008; Aizawa et al., 2013; Mingin et al., 2015) to 100 (Sutherland et al., 1997; Lemack et al., 2000; Jusuf et al., 2001) μl/min. With C57BL6 mice, filling rate in ambulatory filling cystometry seems to be most appropriate at 10 μl/min (Cornelissen et al., 2008; Aizawa et al., 2013; Mingin et al., 2015), because voided volumes are approximately 140–180 μl; similar to that in ambulatory frequency volume charts (Aizawa et al., 2013) (Table 3). The filling rate will affect LUT function. In urodynamic studies, ideally this should be close to the natural filling rate determined by the rate of urine production. However, this can be difficult to do in practize and will lead to long durations between voids. For example, young mice usually urinate around 10 times per day (Aizawa et al., 2013). Natural filling also makes it difficult to calculate the precise bladder infusion volume and is a significant limitation at this small bladder capacity. Thus, artificial filling is usually more practical and advantageous for maximizing urodynamic observations, notwithstanding that mice generally void with longer intervals.

**Table 3:** **Summary of invasive tests for mouse urodynamic studies**.

	**Filling rate (μl/min)**	**Species**	**Sex**	**Weeks**	**Body weight (g)**	**Urodynamic parameters**					
						**Voided volume per micturition (μl)**	**Basal pressure**	**Threshold pressure**	**Micturition pressure**	**Unit**	**Reference**
Conscious	10	C57BL/6J	m	25	40.8 ± 2.0	146 ± 42	7.5 ± 2.8	18.6 ± 4.3	30.3 ± 4.0	cmH_2_O	Aizawa et al., 2013
	10	C57BL/6	m	NA	20–30	140 ± 23.0	NA	17.9 ± 3.0	19.8 ± 1.8	mmHg	Cornelissen et al., 2008
	10		f	NA	20–30	143 ± 28.0	NA	12.2 ± 2.5	19.3 ± 2.4		
	10	Balb/CAN	m	NA	20–30	30.3 ± 4.0	NA	25.5 ± 5.2	19.2 ± 2.8		
	10		f	NA	20–30	40.5 ± 7.3	NA	18.1 ± 3.2	19.0 ± 2.5		
	10	A/J	m	NA	20–30	50.0 ± 4.3	NA	5.3 ± 6.3	17.2 ± 2.6		
	10		f	NA	20–30	51.7 ± 8.4	NA	16.1 ± 3.5	18.4 ± 4.3		
	10	B6:129	m	NA	20–30	164 ± 49.0	NA	16.9 ± 1.8	21.5 ± 3.0		
	10		f	NA	20–30	184 ± 59.0	NA	13.9 ± 2.5	19.7 ± 2.7		
	10	129	m	NA	20–30	50.2 ± 11.9	NA	14.4 ± 1.8	15.8 ± 2.3		
	10		f	NA	20–30	77.5 ± 12.9	NA	10.8 ± 1.4	16.1 ± 1.8		
	41.7	WT (129/SvJ)	m	18–23	NA	240 ± 20	7.1 ± 0.9	17.8 ± 1.4	32.3 ± 2.5	cmH_2_O	Igawa et al., 2004
	41.7		f	18–23	NA	170 ± 10	7.6 ± 0.7	18.8 ± 0.9	33.4 ± 2.4		
	10	C57BL/6	m	6	20–30	about 180	about 8	NA	NA	mmHg	Mingin et al., 2015
	25	Balb/CJ	f	NA	20.2 ± 0.4	110 ± 20	7.11 ± 1.0	12.12 ± 1.22	63.71 ± 5.21	cmH_2_O	Pandita et al., 2000
	25	WT (DBA/1LacJ)	NA	NA	17.2 ± 0.4	49.5 ± 7.6	6.3 ± 2.1	12.5 ± 2.2	36.0 ± 4.5	cmH_2_O	Schröder et al., 2004
	25	MNRI	f	NA	25.7 ± 0.4	150 ± 20	5.9 ± 1.3	9.5 ± 1.0	40.3 ± 3.3	cmH_2_O	Schröder et al., 2003
	25	C57BL/6J	f	NA	15–37	not obtained	NA	about 20	about 40	cmH_2_O	Smith and Kuchel, 2010
	33.3	WT (albino FVB)	m	4–6 mo	NA	200 ± 40	29.9 ± 7.0	42.2 ± 2.5	59.0 ± 5.4	cmH_2_O	Soler et al., 2010
	33.3		f	18 mo	NA	170 ± 60	29.9 ± 2.0	47.3 ± 2.1	64.5 ± 15.7		
	15	WT (129Sv and C57BL/6J)	f	12	24.3 ± 0.9	130 ± 10	NA	NA	34.9 ± 1.6	mmHg	Chen et al., 2005
Restrained	10	C57BL/6N	f	9	18–20	50 ± 20	8.1 ± 1.3	NA	32.0 ± 5.0	cmH_2_O	Kadekawa et al., 2016
	25	C57BL/6	f	8–10	NA	about 20	NA	NA	NA	mmHg	DePaul et al., 2015
Anesthesia	20	Balb/CAN	m	NA	18–22	58 ± 13	NA	NA	12 ± 5	mmHg	Beamon et al., 2009
	13.3	129S1/SvImJ	m	10–12	27.1 ± 0.8	123.6	NA	NA	39 ± 1.4	mmHg	Bjorling et al., 2015
	13.3		f	10–12	21.8 ± 0.8	196.8 ± 28	NA	NA	51.5 ± 2.4		
	13.3	C57BL/6J	m	10–12	27.6 ± 0.5	42.0	NA	NA	21.9 ± 1.5		
	13.3		f	10–12	19.2 ± 0.2	32 ± 2.7	NA	NA	27.1 ± 1.2		
	13.3	NOD/ShiLtJ	m	10–12	29.1 ± 1.3	90.0	NA	NA	25 ± 1.8		
	13.3		f	10–12	24.5 ± 0.4	148 ± 18.7	NA	NA	31.5 ± 2.3		
	13.3	CAST/EiJ	m	10–12	16.6 ± 0.3	111.1	NA	NA	25.4 ± 3.1		
	13.3		f	10–12	13.4 ± 0.6	77.3 ± 10.7	NA	NA	22.8 ± 1.2		
	20	C57BL/6	m	10–12	NA	48	6.9 ± 0.5	4.2 ± 0.2	26.6 ± 1.0	cmH_2_O	Boudes et al., 2011
	25	Balb/CAN	f	NA	26.8	57.5	NA	NA	26.8 ± 2.4	mmHg	Comiter and Phull, 2012
	100	WT (ICR and C57 strains)	f	4–6 mo	32.3	260 ± 110	NA	NA	NA	mmHg	Lemack et al., 2000
	25	C57BL/6	f	2 mo	about 18	about 50	about 12	about 40	48 ± 2.5	cm/W	Smith et al., 2012
	25			12 mo	about 26	about 100	about 8	about 30	47 ± 1.2		
	25			22 mo	about 26	about 120	about 6	about 30	44 ± 1.7		
	25			26 mo	about 30	about 200	about 4	about 25	44 ± 2.3		
	20	C57BL/6J	f	11–13	NA	about 80	about 3	NA	about 40	cmH_2_O	Franken et al., 2016
	80	WT (FVB/N)	m	4–9 mo	24.3–37.7	NA	NA	NA	25.6 ± 4.8	mmHg	Streng et al., 2002a
	80	WT (FVB/N)	m	7–8 mo	30.6 ± 3.3	NA	NA	NA	28.5 ± 5.6	mmHg	Streng et al., 2002b
	10	WT (C57BL6)	m	9–17	25.7 ± 1.1	about 200	NA	NA	NA	cmH_2_O	Birder et al., 2002
	100	WT (129Sv and C57BL/6)	f	NA	18.7	420 ± 210	NA	NA	59.5 ± 12.8	cmH_2_O	Sutherland et al., 1997
	25	C57BL/6J	f	NA	15–37	90 ± 20	NA	about 40	about 60	cmH_2_O	Smith and Kuchel, 2010
Decerebrated	10	WT (C57BL/6Cr)	m	8–12	22.1 ± 0.3	131 ± 11	NA	3.8 ± 0.4	23.0 ± 2.2	mmHg	Yoshiyama et al., 2015

*f, female; m, male; NA, not assessed; mo, months; WT, wild type*.

Of the range of available anesthesic agents, urethane is usually used in mouse cystometry (Sutherland et al., 1997; Jusuf et al., 2001; Birder et al., 2002; Smith and Kuchel, 2010; Boudes et al., 2011; Smith et al., 2012; Bjorling et al., 2015). Focusing on C57BL6 mice, Table 3 shows that the voided volume of conscious mice is 140–146 μl (Cornelissen et al., 2008; Aizawa et al., 2013), and those anesthetized with urethane is 32–90 μl (Smith and Kuchel, 2010; Boudes et al., 2011; Bjorling et al., 2015; Franken et al., 2016). These findings indicate that anesthesia might reduce the voided volume of mice during filling cystometry, although other experimental manipulations may confound this interpretation: For example anesthesia allows a stable trace to be recorded without artifact from physical activity (Smith and Kuchel, 2010; Boudes et al., 2011; Smith et al., 2012; Boudes et al., 2013; Yu et al., 2014; Bjorling et al., 2015; Franken et al., 2016). However, the effects of anesthetic agents on urodynamic parameters cannot be avoided. Urethane was reported to influence the voiding function of rodents less than other agents (Matsuura and Downie, 2000). However, some studies indicated that any form of anesthesia can affect the cystometric patterns of mice (Smith and Kuchel, 2010) in a similar way to rats (Yoshiyama et al., 1993a,b; Aizawa et al., 2015b). Some previous studies using rats indicated that urethane anesthesia can suppress the bladder micturition reflex and NVCs (Cheng and de Groat, 2004). Another study using a decerebrated rat model suggested that urethral activity, which is essential for efficient voiding, is more sensitive to the dose dependent suppressive effect of urethane than afferent or efferent mechanisms controlling the bladder. Furthermore, the afferent limb had a higher sensitivity to urethane than the efferent limb in the micturition reflex pathway. Because effects of urethane persisted after removal of the forebrain, they were presumed to be mediated by actions on the brain stem, spinal cord, or peripheral nervous system (Yoshiyama et al., 2013).

NVC activity has been observed in both awake and anesthetized mice (Pandita et al., 2000; Birder et al., 2002; Cornelissen et al., 2008; Comiter and Phull, 2012; Smith et al., 2012; Mingin et al., 2015; Yoshiyama et al., 2015). It has been proposed that this activity contributes to the volume sensory process, via activation of mechanoreceptors during filling (Lagou et al., 2006b; Streng et al., 2006). NVCs are described as phasic increases of intravesical pressure during filling cystometry, not associated with passage of urine. For precise confirmation of NVC, it is necessary to confirm there is no urine expulsion from the urethra when NVC activity is documented in the bladder pressure recordings. Many previous reports have used a minimum amplitude for NVCs of 5–10 mmHg (Birder et al., 2002; Cornelissen et al., 2008; Comiter and Phull, 2012; Yoshiyama et al., 2015; Kadekawa et al., 2016). However, there are no reliable and well-acceptable diagnostic criteria to define NVCs, which leads to variation of NVC parameters between studies (Pandita et al., 2000; Birder et al., 2002; Cornelissen et al., 2008; Comiter and Phull, 2012; Smith et al., 2012; Mingin et al., 2015; Yoshiyama et al., 2015; Kadekawa et al., 2016).

##### Outlet

The functional examination of the urethral sphincter is difficult in mouse urodynamic studies. Electromyography (EMG) of the external urethral sphincter (EUS) is technically challenging because of their small size (Andersson et al., 2011). Therefore, co-ordinated activity of bladder and urethra under normal and pathological conditions has not been well-characterized in mice, in contrast to numerous studies in rats (Sadananda et al., 2011). There are a few reports that measure the EUS-EMG in the awake mice under restrained conditions. In these, fine wire EMG electrodes were placed percutaneously into or near the EUS and simultaneous measurements of intravesical pressure and EUS-EMG activity were performed during continuous cystometrograms (DePaul et al., 2015; Kadekawa et al., 2016). These reports showed low-amplitude tonic EUS-EMG activity between voids during continuous infusion (DePaul et al., 2015). However, they mainly focused on EUS-EMG activity during the voiding phase, so the specific details during the storage phase is still incompletely described (DePaul et al., 2015; Kadekawa et al., 2016).

##### Sensory function during filling

To investigate afferent pathways from the bladder, direct measurement of afferent nerve activity is necessary and pelvic nerve activity is most often measured. Afferent nerve activity of mice has mainly been measured with *in vitro* experiments (Daly et al., 2007, 2010, 2014; Collins et al., 2013; Mingin et al., 2015) and *in vivo* observations are fewer (Zvara et al., 2010) as *in vivo* measurement is technically difficult. The above studies were performed to investigate the afferent activity during bladder filling, not during the voiding phase as these preparations were not voiding. With *ex vivo* experiments, the urinary bladder and urethra were usually dissected with postganglionic nerves, major pelvic ganglia, and pelvic nerves and placed in a recording chamber recirculated with gassed (95% O_2_ and 5% CO_2_) saline solution at 35 (Daly et al., 2007, 2010, 2014; Collins et al., 2013) or 37 (Mingin et al., 2015) °C. Subsequently, the pelvic nerves were teased into fine branches and placed onto platinum electrodes for recording.

During the storage phase, ramp bladder distension caused an increase in afferent discharge with increasing intravesical pressure (Daly et al., 2007, 2010, 2014; Collins et al., 2013; Mingin et al., 2015). The relationship between afferent discharge and pressure is non-linear. Afferent activity increases markedly when intraluminal pressure is raised between 0 and 20 mmHg, with a smaller increase as intraluminal pressure is further raised between 20 and 45 mmHg) (Daly et al., 2007).

In general, direct measurement of pelvic nerve activity records afferent activity from many units, that includes neurones innervating not only the bladder, but also the urethra and other pelvic organs such as the rectum. To overcome this problem, single-unit mechanosensitive afferent measurement *in vivo* (Aizawa et al., 2012, 2014, 2015a) and *ex vivo* (Ito et al., 2016) in a rat model has been developed and were able to distinguish between A-delta and C fibers. To date the same approach has not been reported for mouse preparations.

#### Pressure flow studies (voiding phase)

##### Bladder

Pressure flow studies are used to assess several urodynamic parameters, including maximum detrusor pressure (P_det max_), maximum flow rate (Q_max_), detrusor pressure at maximum flow rate; (P_det Qmax_), high frequency oscillations of P_det_ and post voided residual volume.

The small voided volume of mice makes difficult the precise time-dependent correlation of Pdet changes and urine flow rate in a pressure-flow study. However, some studies have estimated flow rate during voiding, for example, for example by dividing total voided volume by total flow to obtain average urine flow rate (Smith et al., 2012). Urine flow rate has also been measured with an ultrasonic flow probe surrounding the distal urethra between the rhabdosphincter and penile bulb and connected to a flow meter (Streng et al., 2002a,b). These studies indicated that the maximum urine flow rate of mice was 16.8–17.7 and the average was 1–2.5 ml/min (Streng et al., 2002a,b; Smith et al., 2012).

In mice, the measurement of residual urine volume is also technically challenging, but some studies report values of 2–60 μl in normal conscious mice during urodynamic testing (Pandita et al., 2000; Schröder et al., 2003; Igawa et al., 2004; Chen et al., 2005; Soler et al., 2010). In attempting to measure precise residual urine volume in mice, a single cycle cystometry technique may be employed, where the bladder is emptied by aspiration after voiding. This technique may allow residual urine volume to be measured directly.

Finally, the voiding pressure of mice anesthetized with urethane is significantly higher than that of mice without anesthesia (Smith and Kuchel, 2010). To our knowledge, the effects of anesthesia on residual urine volume has not been measured thus far, but in rats, urethane, and chloral hydrate anesthesia led to less efficient voiding and increased residual urine volume (Streng et al., 2006).

##### Outlet

Technical challenges with flow measurement mean that assessment of voiding in mice is based on the observation of urine output and increased bladder pressure. However, this has the limitation that co-ordinated voiding driven by the pontine micturition center (PMC) cannot be distinguished from non-voiding urine flow (leakage of urine equivalent to detrusor overactivity incontinence).

Mouse cystometric studies have observed intravesical pressure oscillations that accompany urine flow, attributed to high frequency opening and closing of the urethra. This characterization is not seen in other species, except rats (Streng et al., 2002a,b). At the onset of the voiding contraction, the EUS-EMG activity increases in amplitude and shows “bursting” coincident with rapid intravesical pressure oscillations in the cystometric tracing. Subsequently EUS-EMG activity declines after the peak of the contraction coinciding with the void (DePaul et al., 2015). In clinical urodynamics, such an intravesical pressure oscillations with concomitant involuntary urethral sphincter activation implies detrusor sphincter dyssynergia (DSD), as seen in upper spinal or brainstem neurological conditions, such as spinal cord injury or multiple sclerosis.

However, the details of EUS function during bladder contraction is still unclear in mice. EUS-EMG recordings are better-characterized in rats, and exhibit tonic activity before the onset of voiding and bursting activity during voiding, which generates high frequency pressure oscillations. Here a subsequent post-void pressure increase occurs as the bladder remains contracted whilst the EUS ceases bursting and resumes tonic firing (Sadananda et al., 2011, 2013).

##### Sensory function during voiding

There are no published reports of afferent activity measurement during the voiding phase. Pressure-flow studies using a decerebrated arterially-perfused rat show characteristic pelvic nerve recordings during the voiding phase, with bursts of activity corresponding to changes in bladder pressure and EUS activity (Sadananda et al., 2011).

### Special tests

#### Sensation

To date, there are no reports in mice characterizing sensations during the micturition cycle. To know how a mouse senses such activity is not possible (Parsons and Drake, 2011), however functional brain imaging might give a surrogate indication. In rats, there are some functional brain imaging studies using techniques such as functional magnetic resonance imaging (fMRI) and positron emission tomography (PET). These have identified brain regions activated during bladder filling and voluntary control of micturition (Tai et al., 2009; Deruyver et al., 2015; Wong et al., 2015). During storage, the periaqueductal gray (PAG) is activated by afferent input from the urinary bladder, whilst the PMC is inactive (Tai et al., 2009). Storage is also accompanied by activation of several regions including the motor cortex, somatosensory cortex, cingulate cortex, retrosplenial cortex, thalamus, putamen, insula, and the septal nucleus (Tai et al., 2009).

These techniques have also identified in rats brain regions activated during bladder contractions (Tai et al., 2009; Deruyver et al., 2015; Wong et al., 2015). When bladder volume increased to the micturition threshold, the switch from storage to micturition was associated with PMC activation and enhanced PAG activity (Tai et al., 2009). Micturition was also associated with increased activity of several regions including: The motor cortex, thalamus, putamen, cingulate, insula, hypothalamus, substantia nigra, globus pallidus, hippocampus, and inferior colliculus (Tai et al., 2009). A small-animal PET imaging study demonstrated that volume-induced voiding and isovolumetric bladder contractions in rats provoke changes in brain metabolism, including activation of the insular and cingulate cortices, which is consistent with their proposed role in mapping bladder afferent activity (Deruyver et al., 2015). However, knowledge of the precise functions these various regions play in the micturition remains unclear.

## Proxies for disease conditions

A large range of mouse models has contributed to the understanding of pathophysiologies underlying LUTS (Parsons and Drake, 2011). Most are animal models with induced dysfunction, in which relevant pathological changes are experimentally applied to a healthy animal. In addtion, genetically modified mouse models, for instance transgenic mice, have been used to investigate functional urological changes. In this section, we review currently available pathophysiological mouse models and describe their use and also their limitations when interpreting results from animal models to understand human conditions.

### Sensory stimulation models

Animal models of OAB and bladder pain syndrome (BPS) are commonly made by eliciting bladder hypersensitivity and/or inflammation, which is chemically induced by intravesical or intraperitoneal instillation of a noxious substance (Parsons and Drake, 2011). Chemical agents used for this purpose include cyclophosphamide (Boudes et al., 2013), acetic acid (Yoshiyama et al., 2008, 2010), or hydrogen peroxide (Homan et al., 2013; Dogishi et al., 2015).

OAB is a symptom-based diagnosis, so it is currently impossible to develop a true animal model for OAB (Parsons and Drake, 2011). BPS has an important symptomatic element, but there are additional structural changes which could potentially be modeled (Offiah et al., 2016). For example, recent papers indicate that Hunner-type interstitial cystitis shows histologically obvious inflammation (Maeda et al., 2015; Akiyama et al., 2016). An experimental autoimmune cystitis model induced by uroplakin 3A-derived immunogenic peptide might be a possible BPS model in mice. This model showed autoimmune inflammatory changes of the bladder, and key phenotype features (Izgi et al., 2013).

### Reduced compliance models

Spinal cord injury (SCI) models are one of the commonest neurological models used. With SCI models using mice, complete transection of the thoracic spinal cord at vertebral level T8 (DePaul et al., 2015), T8/9 (McCarthy et al., 2009; Kadekawa et al., 2016), or T10 (Wilson et al., 2005) have been reported. The timing of measurements after development of SCI vary substantially from study to study—from 1–2 weeks (McCarthy et al., 2009), 1–6 weeks (Wilson et al., 2005), 4 weeks (Kadekawa et al., 2016), up to 18 weeks (DePaul et al., 2015). SCI is associated with increased bladder weight [2.3 times (Wilson et al., 2005) larger than that of controls]. Cystometric changes after SCI show increased numbers of NVCs (McCarthy et al., 2009; Kadekawa et al., 2016), decreased voiding efficiency (Kadekawa et al., 2016), and increased residual urine volume (DePaul et al., 2015; Kadekawa et al., 2016). The combination of pressure-flow studies and EMG measurements in mice with SCI indicate development of DSD (DePaul et al., 2015) and decreased EUS activity (Kadekawa et al., 2016).

Mice with experimental autoimmune encephalomyelitis have been used to study neuro-inflammatory bladder dysfunction equivalent to that which occurs with multiple sclerosis, and showed detrusor overactivity in severe experimental autoimmune encephalomyelitis (Franken et al., 2016). Extrapolating data from these models has to take into account the uncertainties of transferring findings from an acute model to a chronic human condition. Experimental autoimmune encephalomyelitis is also a transient self-limiting condition that often recovers spontaneously, whereas multiple sclerosis is chronic and progressive.

### Bladder outlet obstruction (BOO) models

Effects similar to BOO in humans are apparently straightforward to model in animals. This has been achieved by partial obstruction of the urethra using some form of ligature. The time to when measurements are made after development of BOO vary from study to study—from 1 week (Schröder et al., 2003), 4 weeks (Austin et al., 2004), 5 weeks (Pandita et al., 2000) up to 6 weeks (Beamon et al., 2009; Comiter and Phull, 2012) following BOO surgery. These mouse models show many of the structural and physiological bladder wall changes seen in human BOO, including muscle cell hypertrophy (Austin et al., 2004; Beamon et al., 2009; Comiter and Phull, 2012), altered responsiveness to stimuli (Austin et al., 2004), and altered spontaneous myogenic activity (Beamon et al., 2009) with enhancement of NVCs (Pandita et al., 2000; Comiter and Phull, 2012).

Many of the published studies using partial BOO murine models have used female mice (Pandita et al., 2000; Schröder et al., 2003; Comiter and Phull, 2012), which complicates their interpretation, given that they are derived to model male benign prostatic hyperplasia (BPH). Furthermore, induced BOO is much more acute and potentially more severe than BPH–particularly if the urethra is ligated by a suture tied firmly against a rigid rod. BOO models showed increased bladder weight 2.5–3.9 times (Pandita et al., 2000; Austin et al., 2004; Beamon et al., 2009; Comiter and Phull, 2012) larger than that of control. BOO murine models may involve not only outlet obstruction but also detrusor insufficiency but no urodynamic features to distinguish have been agreed. Notwithstanding, partial BOO appears to be a good model to study LUTS as it can be reliably reproduced.

### Transgenic models

Knock-out (KO) models and other transgenic animals are used to study and understand the molecular mechanisms involved in both normal LUT physiology and dysfunction. The mouse is most commonly employed, given that it is widely available, easy to maintain and has a relatively short generation interval.

Several transgenic mouse models have been studied in the functional urological field and urodynamic studies have been performed in mice with KO of; alpha_−1D_ receptors (Chen et al., 2005), muscarinic, M2 and M3 receptors (Igawa et al., 2004), purinergic receptor (P2X_3_) (Cockayne et al., 2000), EP1 receptors (Schröder et al., 2004), TRPV_1_ and TRPV_4_ channels (Birder et al., 2002; Yoshiyama et al., 2015), neuronal nitric oxide synthase (nNOS) (Sutherland et al., 1997), inducible nitric oxide synthase (iNOS) (Lemack et al., 2000), uroplakin II (UPII) and III (UPIIIa) (Aboushwareb et al., 2009), Ncx/Hox11L.1 (a member of the Hox11 homeobox gene family) (Jusuf et al., 2001). Studies have also described Immp2l^Tg(Tyr)979Ove^ mutant mice with mitochondrial dysfunction which have a deficiency of Immp2l protein (Soler et al., 2010), and aromatase overexpressing transgenic mice (Streng et al., 2002a,b). Specific urodynamic features seen in these mice, which informed the phenotype and pathophysiological understanding have been reviewed elsewhere (Parsons and Drake, 2011).

### Aging models

An appropriate study to clarify the pathophysiology of aging is needed but there are many limitations to performing such an investigation. In the elderly human population there are important indirect interactions due to comorbidities such as hypertension, diabetes mellitus, hyperlipidemia, and cerebrovascular or cardiovascular diseases. It is also difficult to separate the influence of such disorders from that of aging. In rodent models, the influence of at least some of these comorbidities can be avoided though they are rarely evaluated and reported. The age of mice used for aging studies has varied from 12 months (Lai et al., 2007), 70 weeks (Shenfeld et al., 2005), 20 months (Perše et al., 2013), 22 months (Smith et al., 2012), 18–24 months (Lagou et al., 2006a), 24 months (Daly et al., 2014), 26 months (Smith et al., 2012), and 28–34 months (Lagou et al., 2006b).

To our knowledge, there is only one study that investigated age-related changes to mice cystometric parameters (Smith et al., 2012). This showed that aging (26 months) is associated with an impaired ability to respond to the challenge of continuous bladder filling with cyclic voiding and diminished bladder volume sensitivity (Smith et al., 2012).

In addition, organ bath studies using isolated mice bladder show that both micromotion related activity and the phasic component of the contractile response to muscarinic agonists are substantially reduced in aging mice (Lagou et al., 2006b). Another immunohistochemical analysis showed that older mice showed patchy denervation of the detrusor (Lagou et al., 2006a).

### Other models

Mingin and colleagues demonstrated that some social stress decreased voided volume in young male mice, and they proposed this approach as an overactivity model by social stress (Mingin et al., 2015). Acute exposure of part of the skin to cold stimuli can also evoke rapid bladder contractions and voids in anesthetized mice and was proposed as an acute cold-induced urgency model (Uvin et al., 2015). These models lead to increased sensory activity, which is a mechanism that has been proposed as a potential cause of urgency.

## Conclusions

Animal experiments are fundamental for understanding LUT function and these experiments also contribute to investigation of pathophysiological changes. Rats are currently the most used animal model for neuro-urodynamic studies, and mouse urodynamics has not been so well-characterized. However, the mouse models available, notably genetic modifications, provide valuable mechanistic insights. The present review covers recent knowledge of mouse urodynamics and considers potential implications in the clinical field. However, there are some differences between mouse and human urodynamics which must be considered when neuro-urodynamics to interpret voiding in mice models. Available disease models in mice have crucial issues of interpretation when deriving translational value. Nonetheless, mouse urodynamic tests should give important information on bladder physiology, pathophysiology and pharmacology.

## Author contributions

Substantial contributions to the conception or design of the work; HI, AP, YI, AK, CF, and MD. The acquisition of the data; HI, YI, and AK. The analysis, or interpretation of data for the work; HI, AP, YI, AK, CF, and MD. Drafting the work or revising it critically for important intellectual content; HI, AP, YI, AK, CF, and MD. Final approval of the version to be published; HI, AP, YI, AK, CF, and MD. Agreement to be accountable for all aspects of the work in ensuring that questions related to the accuracy or integrity of any part of the work are appropriately investigated and resolved; HI, AP, YI, AK, CF, and MD.

### Conflict of interest statement

The authors declare that the research was conducted in the absence of any commercial or financial relationships that could be construed as a potential conflict of interest.

## Abbreviations

BOO: bladder outlet obstruction
BP: basal pressure
BPH: benign prostatic hyperplasia
BPS: bladder pain syndrome
DSD: detrusor sphincter dyssynergia
EMG: Electromyography
EUS: the external urethral sphincter
FVC: frequency-volume chart
LUTS: lower urinary tract symptom
NVCs: non-voiding contractions
OAB: overactive bladder
PAG: periaqueductal gray
PMC: pons micturition center
SCI: Spinal cord injury
TP: threshold pressure
VSA: voiding spot assays.
